# Post-Blink Blur Time—A Simple Test to Detect Dry Eye-Related Visual Disturbances

**DOI:** 10.3390/jcm13092473

**Published:** 2024-04-24

**Authors:** Igor Petriček, Dina Lešin Gaćina, Martina Tomić, Tomislav Bulum, Iva Bešlić, Sania Vidas Pauk

**Affiliations:** 1 Department of Ophthalmology, Zagreb University Hospital Center, 10000 Zagreb, Croatia; 2 School of Medicine, University of Zagreb, 10000 Zagreb, Croatia; 3 Department of Diabetes and Endocrinology, Vuk Vrhovac University Clinic for Diabetes, Endocrinology and Metabolic Diseases, Merkur University Hospital, 10000 Zagreb, Croatia

**Keywords:** post-blink blur time, dry eye disease, visual function, diagnostic tests, ocular surface, tears

## Abstract

**Background**: Dry eye disease (DED) stands out as one of the most common eye conditions encountered in clinical settings. This study aimed to determine the diagnostic ability and feasibility of post-blink blur time (PBBT) in detecting patients with DED symptoms. **Methods**: The study included 200 subjects, 100 with and 100 without DED symptoms defined by the Schein questionnaire, who underwent assessment of DED signs [visual acuity, PBBT, conjunctival hyperemia, lid-parallel conjunctival folds—LIPCOF, tear film break-up time—TBUT, fluorescein corneal staining, and meibum score]. **Results**: DED subjects had a lower PBBT than controls (*p* < 0.001), with subjective (6 (1–45) s vs. 8 (1–70) s) and objective (6 (2–33) s vs. 8 (2–50) s) PBBT measurements being similar between repeats. The correlations between subjective and objective PBBT measurements were significantly positive (R = 0.873, *p* < 0.001). Subjective PBBT was negatively related to the Schein questionnaire (R = −0.217, *p* = 0.002), conjunctival hyperemia (R = −0.105, *p* = 0.035), and corneal staining (R = −0.153, *p* = 0.031), while positively related to the TBUT (R = 0.382, *p* < 0.001) and meibum score (R = 0.106, *p* = 0.033). Logistic regression analysis showed DED symptoms were significantly associated with subjective PBBT (AOR 0.91, *p* = 0.001), TBUT (AOR 0.79, *p* < 0.001), meibum score (AOR 0.65, *p* = 0.008), LIPCOF (AOR 1.18, *p* = 0.002) and corneal staining (AOR 1.14, *p* = 0.028). **Conclusions**: Subjective self-reported PBBT is a reliable and non-invasive screening test for evaluating time-wise changes in visual acuity and detecting a tear film dysfunction.

## 1. Introduction

Among various eye conditions, dry eye disease (DED) continues to be highly prevalent in clinical practice, with prevalence rates ranging from 5% to 50%. There are significant variations in both signs and symptoms, leading patients to seek eye care due to its pervasive impact on daily life [1,2]. It represents one of the commonly experienced ocular health issues, especially in light of recent shifts in environment and lifestyle influenced by the extensive use of visual display terminals (VDTs), wearing contact lenses, and the aging process [3,4]. Currently, the most broadly accepted definition of dry eye disease was proposed by the Tear Film and Ocular Surface Society International Dry Eye Workshop (TFOS DEWS II) in 2017 [1]. The original TFOS DEWS extended the concept of symptoms to include visual disturbances [5], whereas TFOS DEWS II conceded that discomfort and visual disturbance symptoms should remain integral aspects of DED [1].

The impact of DED on visual acuity adversely affects activities such as driving, reading, and computer use, thereby also leading to adverse effects on psychological well-being. [2,4,6]. These consequences also extend to the workplace, where diminished productivity and compromised work quality result in significant economic losses [2]. Ophthalmologists often underestimate the significance of the effects of DED on vision and its impact on patients’ quality of life related to vision, failing to accord it the necessary importance.

The tear film constitutes the initial component of the ocular surface encountered by light as it travels along the pathway to the retina. [4]. It plays a crucial role in maintaining the stability of ocular optical quality, ensuring a consistently intact and uniform tear film, which is vital for achieving high-quality retinal images. Any deterioration in the tear film can thus negatively impact visual function. Typically, the tear film is naturally unstable and experiences irregular disruptions after a blink, leading to tear film break-up. [7]. Subsequent unevenness in the thickness of the tear film across the ocular surface adversely affects the quality of the eye’s most critical refractive surface [7,8]. Extensive documentation indicates that eyes with a short tear film break-up time (TBUT) experience a notably faster decline in optical quality following a blink compared to normal eyes [9,10]. Consequently, these patients tend to experience a decline in visual acuity over time after keeping their eyes open for a few seconds [6]. A significant consequence of the short TBUT in individuals with DED is an elevated blink rate, which serves as a compensatory mechanism. This increased blink rate stimulates tear secretion and facilitates the generation of a fresh tear film layer on the ocular surface with each blink [11]. In relaxed conditions, individuals with dry eye blink approximately twice as frequently (33.9 blinks per minute) compared to normal controls, who exhibit a lower blink rate of 14.3 blinks per minute [12]. Daily activities such as reading, using VDTs, driving, etc., involuntarily suppress spontaneous blinking, which destabilizes the tear film and prevents its recovery on the eye’s surface, resulting in uncomfortable visual disturbances [2,13].

Although patients with DED frequently report poor visual quality, traditional visual acuity tests often yield clinically normal results and are not sensitive enough to detect visual deterioration related to DED [14,15,16]. The detection of impaired visual acuity in DED is only possible through functional visual acuity testing (FVA) that measures visual acuity after sustained eye opening without blinking for a defined time interval and presents a simulation of the visual function of daily acts of gazing [6]. At present, various tests and methodologies are available for assessing dynamic visual function. These include the SSC-350 FVA measurement system, evaluation of best-corrected visual acuity decay through the inter-blink interval visual acuity decay (IVAD) test, sequential assessments of ocular and corneal higher-order aberrations, and the measurement of dynamic visual quality using the Optical Quality Analysis System [4]. However, the equipment required for these methods is excessively complex and financially inaccessible for routine clinical practice. Both the instruments and the methods themselves are predominantly employed for experimental purposes. The underestimation and lack of awareness regarding the relevance of FVA loss in DED patients, coupled with the absence of readily available testing equipment, often result in a failure to recognize and diagnose DED-associated reductions in visual function accurately. This, in turn, hinders the appropriate diagnosis and treatment of this condition.

The authors of the present work aimed to investigate whether there is a clinically applicable test that might detect visual disturbances in patients with dry eye and be used for routine screening for DED during regular, daily practice. As mentioned earlier, the tear film dysregulates and destabilizes in a particular time interval after a blink, leading to visual impairment. That interval is shorter in dry eye patients than in healthy subjects. The authors hypothesized that the time interval between the last blink and the occurrence of the blurring of the best-line visual acuity on standard Snellen optotypes, self-reported by the patient, might be objectively measured—namely, the post-blink blur time (PBBT). Optical quality in patients with DED deteriorates more rapidly after the blink compared to normal eyes; this test might help in distinguishing dry eyes from healthy ones. The PBBT is not visual acuity testing; it does not evaluate best-corrected visual acuity (BCVA), but rather the decline in BCVA following a sustained deterioration of BCVA after sustained eye-opening without blinking, until the first noticeable blurring of the optotypes in the best line on standard Snellen charts occurs. The test is simple and accessible. It might be performed during a routine ophthalmology examination following visual acuity testing and can be conducted by almost every eye specialist, not significantly prolonging the duration of a standard exam. The testing typically lasts around two to three minutes. Ultimately, it can aid in routine everyday dry eye diagnostics, as a simple, non-invasive screening test that may facilitate timely medical examination and treatment.

This study aimed to investigate the impact of post-blink tear film changes on visual acuity in patients with dry eye symptoms, and those without, by measuring the time from the last blink until the occurrence of blurring of visual acuity using standard Snellen optotypes, known as the PBBT. The objectives were to determine whether the PBBT can detect patients with dry eye symptoms, asses the clinical applicability and feasibility of the PBBT, and explore the correlation between the PBBT and tear break-up time (TBUT), another objective dry eye test, and subjective dry eye symptoms.

## 2. Materials and Methods

### 2.1. Study Design and Subjects

This study was conducted in accordance with the Declaration of Helsinki and approved by the Ethics Committee of the Zagreb University Hospital Center. Before participating, all patients received both written and oral information about the research and willingly signed written informed consent.

The study included 200 participants who consecutively visited the Ophthalmology Department within five months, spanning from May to September 2021 (intending to minimize the potential influence of factors associated with colder weather on the study’s outcomes) and were randomly chosen during the routine clinical activities of the first author. During the screening visit, participants provided informed consent, and the first author obtained a case history using a standardized Schein questionnaire, tailored for this research to assess the severity of dry eye symptoms [17]. 

The subjects were divided into two groups based on the reported dry eye symptoms: the dry eye group (*n* = 100) comprising individuals with dry eye symptoms (Schein questionnaire score 1–20), and the control group (*n* = 100), which included individuals without dry eye symptoms (Schein questionnaire score 0).

Afterward, all the subjects underwent a standard ophthalmology examination, which included BCVA testing (Pentacam HR; Oculus Inc., Wetzlar, Germany), PBBT measurement, slit lamp examination, conjunctival hyperemia assessment, lid-parallel conjunctival folds (LIPCOF) and TBUT measurements (Biotech, Minneapolis, MN, USA, Fluorescein Sodium Ophthalmic Strip USP), fluorescein surface-staining assessment, and finally Meibomian gland (MG) assessment. A sole examiner, the first author, conducted all the measurements, and these were cross-verified by the last author of the study. Any measurements displaying discrepancies between the two observers were omitted. Inclusion criteria required participants to be 18 years or older, non-contact lens wearers, to exhibit normal findings in other anterior ocular surfaces, and not to use topical ophthalmic medication. The exclusion criteria included a past history of ocular trauma and surgery, acute infections, glaucoma, the presence of other ocular surface diseases or irregularities, systemic diseases or medications that could impact the ocular surface, and insufficient cooperation.

### 2.2. Schein Questionnaire

The clinical parameters assessed included the severity of dry eye symptoms, which were evaluated through the Schein questionnaire [5,17]. The Schein questionnaire was chosen due to its frequent utilization in the authors’ routine clinical practice, providing them with extensive experience. Additionally, the questionnaire has been validated for use in Croatian translation. The first author administered the questionnaire, which consists of six disease-specific items. Patients rate their self-reported symptoms of dry eye on a scale from 0 to 4 (0—none, 1—rarely, 2—sometimes, 3—often, and 4—all the time). The subscale scores on the Schein questionnaire have a potential range of 0 to 24, with higher scores indicating a greater presence of problems or symptoms. This questionnaire is characterized by its simplicity, practicality, brevity, and universal comprehensibility, making it accessible to patients of all ages, including the elderly. Importantly, the Schein questionnaire is not protected by copyright, ensuring widespread availability without restrictions. However, a limitation of this questionnaire is its failure to assess the impact of dry eye symptoms on patients’ vision, daily activities, and overall quality of life. Additionally, it lacks a validated cut-off value for DED, unlike some other questionnaires such as the Ocular Surface Disease Index (OSDI) and the 5-Item Dry Eye Questionnaire (DEQ-5).

### 2.3. Best-Corrected Visual Acuity (BCVA)

BCVA testing was conducted using standard Snellen optotypes at 6 m, starting with the right eye and then on to the left eye.

### 2.4. Post-Blink Blur Time (PBBT)

The PBBT was performed after BCVA testing and before any other invasive tests. It was performed by the patient (subjective self-reported PBBT) and by the first author (objective PBBT). The procedure was repeated three times for both the right and left eyes.

After determining the BCVA for the right eye, the patient was instructed to fixate on the first number of the best visual acuity line on the Snellen chart at a 6 m distance. Then the patient was asked to blink naturally three times and to say “start” upon reopening the examined eye. Subsequently, the patient began subjectively self-measuring the time interval from the last blink until the blurring of the first number in the last line, reporting this as a self-reported PBBT in seconds. At the same time, when the patient said “start”, the examiner started the stopwatch and objectively measured the time interval between the blink and the moment of reported blurring of the first number in the best line. After that, the patient reported the subjective PBBT in seconds. The examiner then stopped the stopwatch and measured the objective PBBT in seconds. If the patient blinked before that point, the testing was repeated for that measurement.

### 2.5. Conjunctival Hyperemia

Conjunctival hyperemia was evaluated using the Cornea and Contact Lens Research Unit (CCLRU) grading scale. [18].

### 2.6. Lid-Parallel Conjunctival Folds (LIPCOF)

LIPCOF was observed without fluorescein, on the bulbar conjunctiva, specifically in the region perpendicular to the temporal limbus [19].

### 2.7. Tear Break-Up Time Test (TBUT)

The tear break-op time (TBUT) was measured using standardized fluorescein strips (Biotech, Fluorescein Sodium Ophthalmic Strip USP) [20]. The process was repeated three times for both the right and left eyes.

### 2.8. Fluorescein Surface Staining

Fluorescein staining of the cornea and conjunctiva was evaluated using the National Eye Institute/Industry Workshop (NEI) scale (National Eye Institute, Bethesda, MD, USA) [21].

### 2.9. Meibomian Gland Assessment

Meibomian gland (MG) assessment involved MG expression (MGX) on the lower eyelid of both eyes, conducted after other dry eye tests to ensure they remained unaffected by MGX. Korb’s technique for the digital expression of the Meibomian glands was employed [22]. The meibum quantity (MG expressibility) was evaluated on a scale as follows: 3—most MG ducts secrete a clear meibum; 2—approximately half of the excretory MG ducts secrete meibum, with a scarce secretion; 1—a few ducts secrete meibum, and the secretion is barely visible; 0—no visible MG secretion after expression. Meibum quality was assessed using the following scale: 4—clear; 3—cloudy; 2—cloudy with debris (granular); 1—toothpaste-like.

### 2.10. Statistical Analysis

A statistical analysis was conducted using StatisticaTM software, version 14.0.1 (TIBCO^®^ Inc., Palo Alto, CA, USA). The normality of the data distribution was assessed using the Kolmogorov–Smirnov test, and variance homogeneity was examined using the Levene test. The descriptive analyses presented the mean ± SD and median (min-max) for continuous data, median (min–max) for ordinal data, and numbers for categorical data. Differences in the distributions of continuous and ordinal data were assessed through the *t*-test and Mann–Whitney test for independent variables and the Friedman ANOVA test for dependent variables. Nonparametric tests were employed when the assumption of homogeneity of variance for the tested variables was not met. The Chi-square test was employed to assess differences in the distributions of categorical data. The Spearman rank correlation test was utilized to evaluate associations between examined variables. Additionally, binary univariate and multiple logistic regression analyses were conducted to determine the strength and independence of associations. For all analyses, the predefined level of statistical significance was set at 0.05.

## 3. Results

The study involved 200 participants, consisting of 45 males and 155 females, with a mean age of 46.28 ± 16.34 years. 

Table 1 provides descriptive statistics for the baseline characteristics of the study participants. Individuals with dry eye symptoms were significantly older than those in the control group (*p* < 0.001), and there was a significantly higher proportion of women in the dry eye group compared to the control group (*p* < 0.001). The median Schein questionnaire score for the dry eye group was 7 (min 1–max 20), while it was 0 for the control group. However, no significant difference in BCVA was observed between the examined groups (*p* = 0.650).

All three subjective self-reported and three objective PBBT measurements for both the right and left eyes were significantly lower in the dry eye group compared to the control group (*p* < 0.001). However, no significant differences were identified within the examined groups concerning the subjective self-reported and objective PBBT measurements for both the right and left eyes (*p* > 0.05) (refer to Table 2 and Table 3). The correlations between the subjective self-reported and objective PBBT measurements of the right and left eye were significantly positive, ranging from very good to excellent (Table 4).

As no significant differences were observed, as determined by the Friedman ANOVA test, between the three measurements of PBBT and TBUT for both the right and left eyes, and using the Wilcoxon test, in scores for other signs of dry eye signs between the right and left eye within the examined groups, further statistical analyses utilized the mean values of both eyes (median, min–max).

The two groups did not show significant differences in conjunctival hyperemia (CCRLU) (*p* = 0.626). However, the LIPCOF was higher and the TBUT was shorter in individuals with dry eye symptoms, compared to those without dry eye symptoms (*p* < 0.001). The fluorescein corneal-staining score for both eyes was higher in individuals with dry eye symptoms than in those without dry eye symptoms (*p* = 0.047).

The meibum quantity score after Meibomian gland expression (MGX) for both eyes was significantly lower in the dry eye group, compared to the control group (*p* < 0.001). However, there was no significant difference between the groups in the meibum quality scores after MGX for both eyes (*p* = 0.205). Unfortunately, these specific data are not presented in the table.

The subjective self-reported PBBT for both eyes exhibited significant negative correlations with the Schein questionnaire score (*p* = 0.002), the presence of conjunctival hyperemia (CCRLU) (*p* = 0.035), and the fluorescein corneal-staining score for both eyes (*p* = 0.031). Conversely, it showed significant positive correlations with the TBUT score for both eyes (*p* < 0.001) and the meibum quantity score after Meibomian gland expression (MGX) for both eyes (*p* = 0.033). No significant correlation was observed between the subjective self-reported PBBT and the LIPCOF score (*p* = 0.687) and the meibum quality score after MGX for both eyes (*p* = 0.197) (refer to Table 5).

Table 6 provides baseline characteristics, the subjective self-reported PBBT, and other dry eye signs associated with dry eye using binary logistic regression analysis. The strongest associations were identified for older age (OR 1.64, *p* < 0.001) and female gender (OR 4.85, *p* < 0.001). Significant relationships were also observed for a shorter PBBT (OR 0.92, *p* = 0.004), shorter TBUT (OR 0.77, *p* < 0.001), lower meibum quantity score (OR 0.59, *p* < 0.001), higher LIPCOF (OR 1.04, *p* < 0.001), and fluorescein corneal-staining score (OR 1.05, *p* = 0.019). After adjusting for age and gender, all these variables remained independent and significantly associated with dry eye (*p* < 0.05).

## 4. Discussion

For a diagnostic test to be integrated into a routine ophthalmology practice, it must possess a straightforward methodology, be easily accessible and affordable, and not considerably extend the duration of the routine examination. In this study, the authors investigated a simple diagnostic method, PBBT, to assess the impact of post-blink tear film changes on visual acuity by using the standard optotypes. The aim was to determine whether this test could effectively detect patients with dry eye symptoms and distinguish them from healthy subjects, as well as to evaluate whether it could serve as a simple and fast screening test for DED diagnosis. This study’s PBBT assessment took approximately two to three minutes after the ordinary visual acuity assessment. Most patients cooperated effectively, although a few older patients faced challenges either in understanding the examination procedure or maintaining open eyes due to discomfort. Those patients were not included in the study.

The present study’s results showed median self-reported PBBT values to be 6 (1–45) s in patients with dry eye symptoms and 8 (1–70) s in healthy subjects. The values for the objective PBBT using a stopwatch were slightly, but not significantly, shorter in both groups, and they turned out to be 6 (2–33) s and 8 (2–50) s, respectively. The self-reported and objective PBBT values of the right and left eye were significantly lower in the group with dry eye symptoms, and that might be used for reliable discrimination between the patients with and without dry eye symptoms. Interestingly, patients reported a momentary and not gradual blurring of vision, which is shown by the high reproducibility of the results in all three measurements, even for self-reported and objective PBBT. A more significant agreement of both assessments was present in the area of short values, while in the area of extended values, the tendency of patients to report lengthy assessments (a longer time in seconds) compared to the doctor was noticed. These findings were essential, since the results showed that a self-reported PBBT might be used as a reliable method. The patients’ assessments were highly correlated with the examiner’s control values in all three measurements, which allows the use of self-reported PBBTs in routine work and without the need for control with a stopwatch, which would undoubtedly complicate the entire procedure. Indeed, since the results showed minimal differences in PBBT values between the eyes, the authors suggest that a single unilateral measurement of PBBT can detect dry eye symptoms, making the method even more practical.

In this study, the subjective self-reported PBBT demonstrated strong correlations with dry eye symptoms and most other clinical tests, suggesting its objectivity. A significantly strong correlation was noted, especially, between PBBT and TBUT values in all three measurements for both eyes. This suggests that both diagnostic tests assess distinct clinical manifestations of the same physical phenomenon: the instance of tear film destabilization over the corneal surface following a blink. However, PBBT is a non-invasive diagnostic method, while the performance of TBUT interferes with the tear film due to fluorescein installation. Hence, additional research should explore the relationship between PBBT and non-invasive tear break-up time (NIBUT), a technique for measuring tear break-up time that does not require using fluids or dyes and is considered more physiological than TBUT [20].

Further statistical analysis showed that shorter PBBT values were significantly associated with dry eye complaints, regardless of age and gender. These findings underscored the significance of a shorter PBBT value as a significant predictor and indicator of dry eye symptoms.

Given the observed correlation, the PBBT test, designed to identify breaks on the tear film’s surface when it destabilizes over the eye surface, holds promise for dry eye assessment. However, despite the longstanding focus on functional visual acuity in dry eye research, there are limited reports in the literature regarding diagnostic methods akin to PBBT, which specifically address occurrences on the ocular surface and visual acuity during the inter-blink interval. Indeed, there is currently insufficient data available for a comprehensive comparison. Goto et al. measured FVA during sustained eye opening without blinking for 10–20 s after the instillation of topical anesthesia, utilizing the same spectacles worn for ordinary BCVA testing [6]. However, this method has drawbacks, including uncertainty about the timing of FVA measurement, lack of objectivity, and potential interference with the ocular surface due to the application of topical anesthesia. Walker and coworkers developed inter-blink interval visual acuity decay (IVAD), a real-time, computer-based measure of visual acuity decay that involves the identification of the orientation of the Landolt C optotype during the inter-blink interval at an individualized BCVA between blinks and measures parameters based on patient responses, thereby showing the effect of optical aberrations on visual function indirectly [23]. The IVAD test is performed without anesthesia; however, it is essential to mention that this diagnostic tool is patented and cannot be used without permission. It necessitates a particular computer program and is time-consuming, making it unsuitable for routine clinical use. In the Osaka Study, the Functional Visual Acuity Measurement System (Kowa, Aichi, Japan) was employed to assess the continuous change in visual acuity over time among a population of visual display terminal (VDT) users. FVA was defined as the mean value of time-wise changes in visual acuity during the examination and was measured over 60 s under daily vision correction (not with the best-corrected visual acuity), without the use of topical anesthesia. During the measurement period, subjects were allowed to blink naturally. Participants indicated the orientation of automatically presented Landolt rings by manipulating a joystick. The results showed no significant differences between the dry eye group and the controls regarding FVAs, indicating that the FVA parameter of the presented system is not effective enough in DED prediction. The diagnostic accuracy of that system was enhanced with the combination of FVA measurement with a subjective eye symptoms questionnaire. In general, based on the recent study, the interchangeability of dry eye diagnostic platforms is limited, and when interpreting non-invasive tear film values, it is important to consider the unique characteristics of each instrument. Furthermore, the elevated coefficient of variation observed in patients with DED, compared to those without it, highlights the inherent variability in tear film function, regardless of the specific diagnostic device utilized [24].

## 5. Limitations

Certainly, there are potential limitations to acknowledge in this study. Firstly, the Schein questionnaire, utilized to assess dry eye symptoms, does not specifically evaluate the impact of these symptoms on subjects’ vision, daily activities, and overall quality of life. In contrast, certain other questionnaires, such as the Ocular Surface Disease Index (OSDI), address these broader aspects of the patient experience. The choice of the Schein questionnaire in this study was based on the authors’ familiarity with its use in clinical practice, their experience with its implementation, and its lack of copyright protection. While other questionnaires may address a broader range of factors, the practicality and accessibility of the Schein questionnaire influenced its selection.

Additionally, it is important to note that this study introduces a novel method, and there are no comparable diagnostic tests documented in the literature for direct comparison. This uniqueness contributes to the limited availability of similar tests for reference.

## 6. Conclusions

The PBBT has been confirmed as an objective, reliable, and non-invasive screening test in evaluating time-wise changes in visual acuity and detecting tear film dysfunction. Furthermore, the method presented in this study appears to be suitable and cost-effective for routine ophthalmology practice. Its implementation does not significantly extend or complicate a standard ophthalmic examination, and it does not require any additional or expensive equipment or devices. Because of the significant correlation, a short PBBT, along with the presence of symptoms and other clinical signs of dry eye, could potentially improve the accuracy of DED diagnosis. However, since the method requires a patient’s cooperation, it may not be suitable for patients with cognitive impairments or the elderly, who have difficulties in understanding the instructions. Despite potential limitations, the PBBT is deemed a valuable diagnostic tool for identifying visual acuity impairment in individuals with dry eye. This can contribute to a prompt medical examination and the development of tailored treatment plans for these patients. Thus, further investigations with a larger number of observers, subjects, and centers should be conducted to validate this study’s findings.

## Figures and Tables

**Table 1:** Baseline characteristics of subjects included in the study.

	Dry Eye Group (*n* = 100)	Control Group (*n* = 100)	t ^a^ Chi ^b^	*p*
Age (years) *	51.17 ± 15.16	41.39 ± 16.08	4.426 ^a^	<0.001 ^a^
Gender (m/f) **	10/90	35/65	17.921 ^b^	<0.001 ^b^
Schein questionnaire *	7.32 ± 4.31	0.00	19.690 ^a^	<0.001 ^a^
BCVA of both eyes (decimal) *	0.99 ± 0.05	0.99 ± 0.07	0.454 ^a^	0.650 ^a^

* *x* ± SD, ** numbers, ^a^ *t*-test, df = 198, ^b^ Chi-square test, df = 1. Abbreviations: BCVA: best-corrected visual acuity.

**Table 2:** Three subjective self-reported PBBT measurements of both eyes in subjects divided into two groups, according to the presence/absence of dry eye symptoms.

	Dry Eye Group (*n* = 100)	Control Group (*n* = 100)	Z ^a^	*p* ^a^
1. PBBT of the right eye (s)	6 (2–43)	9 (3–70)	−3.945 ^a^	<0.001 ^a^
2. PBBT of the right eye (s)	6 (2–45)	8 (2–56)	−3.413 ^a^	<0.001 ^a^
3. PBBT of the right eye (s)	6 (2–40)	8 (2–60)	−3.861 ^a^	<0.001 ^a^
1. PBBT of the left eye (s)	6 (2–43)	8 (2–60)	−4.397 ^a^	<0.001 ^a^
2. PBBT of the left eye (s)	5 (1–23)	8 (1–58)	−4.627 ^a^	<0.001 ^a^
3. PBBT of the left eye (s)	6 (1–30)	7.5 (1–50)	−4.012 ^a^	<0.001 ^a^
Chi ^b^	9.520 ^b^	7.193 ^b^		
Pb	0.090 ^b^	0.207 ^b^		

Med (Min–Max), a Mann–Whitney test, b Friedman ANOVA test df = 5. Abbreviations: PBBT: post-blink blur time.

**Table 3:** Three objective PBBT measurements of both eyes in subjects divided into two groups, according to the presence/absence of dry eye symptoms.

	Dry Eye Group (*n* = 100)	Control Group (*n* = 100)	Z ^a^	*p* ^a^
1. PBBT of the right eye (s)	6 (2–33)	8 (3–50)	−4.388 ^a^	<0.001 ^a^
2. PBBT of the right eye (s)	6 (2–33)	7.5 (2–40)	−3.497 ^a^	<0.001 ^a^
3. PBBT of the right eye (s)	6 (2–30)	7 (2–40)	−3.565 ^a^	<0.001 ^a^
1. PBBT of the left eye (s)	5 (2–31)	8 (2–43)	−4.556 ^a^	<0.001 ^a^
2. PBBT of the left eye (s)	5 (1–18)	7 (2–30)	−4.498 ^a^	<0.001 ^a^
3. PBBT of the left eye (s)	5 (1–25)	6.5 (1–30)	−4.062 ^a^	<0.001 ^a^
Chi ^b^	10.323 ^b^	6.954 ^b^		
Pb	0.089 ^b^	0.246 ^b^		

Med (Min–Max), a Mann–Whitney test, b Friedman ANOVA test df = 5. Abbreviations: PBBT: post-blink blur time.

**Table 4:** Correlations between subjective self-reported and objective PBBT measurements of both eyes in all subjects included in the study.

		Objective PBBT Measurements					
		1. Right Eye	2. Right Eye	3. Right Eye	1. Left Eye	2. Left Eye	3. Left Eye
Subjective self-reported PBBT m	1. right eye	20.856 *	0.689 *	0.634 *	0.564 *	0.541 *	0.521 *
	2. right eye	0.710 *	0.879 *	0.732 *	0.633 *	0.628 *	0.551 *
	3. right eye	0.673 *	0.708 *	0.874 *	0.595 *	0.574 *	0.529 *
	1. left eye	0.604 *	0.617 *	0.545 *	0.880 *	0.758 *	0.703 *
	2. left eye	0.583 *	0.619 *	0.577 *	0.783 *	0.891 *	0.751 *
	3. left eye	0.548 *	0.543 *	0.546 *	0.767 *	0.764 *	0.859 *

* *p* < 0.001 Spearman rank correlation test.

**Table 5:** Correlations between the subjective self-reported PBBT values, dry eye symptoms, and dry eye signs values of both eyes in all subjects included in the study.

Subjective Self-Reported PBBT of Both Eyes (Seconds)			
	Spearman R	t(N-2)	*p*
Schein questionnaire score	−0.217	−3.132	0.002
CCRLU of both eyes	−0.105	−2.111	0.035
LIPCOF of both eye	0.020	0.403	0.687
TBUT of both eyes	0.382	14.294	<0.001
Fluorescein corneal staining of both eyes	−0.153	−2.176	0.031
Meibum quantity score of both eyes	0.106	2.134	0.033
Meibum quality score of both eyes	0.065	1.291	0.197

Abbreviations: PBBT: post-blink blur time; CCRLU: Cornea and Contact Lens Research Unit; LIPCOF: lid-parallel conjunctival folds; TBUT: tear break-up time.

**Table 6:** Predictors and indicators of dry eye symptoms using logistic regression analysis.

	OR (95%CI)	*p*	AOR (95%CI) *	*p* *
Age (years)	1.64 (1.09–2.48)	<0.001	/	
Gender (female)	4.85 (2.23–10.54)	<0.001	/	
Subjective self-reported PBBT of both eyes	0.92 (0.87–0.97)	0.004	0.91 (0.86–0.96)	0.001
CCRLU of both eyes	0.96 (0.59–1.62)	0.057	1.03 (0.57–2.07)	0.229
LIPCOF of both eyes	1.04 (1.02–1.06)	<0.001	1.18 (1.04–1.54)	0.002
TBUT of the right eye	0.77 (0.69–0.86)	<0.001	0.79 (0.71–0.89)	<0.001
Fluorescein corneal staining of both eyes	1.05 (1.01–1.24)	0.019	1.14 (1.09–1.48)	0.028
Meibum quantity score of both eyes	0.59 (0.44–0.79)	<0.001	0.65 (0.47–0.89)	0.008
Meibum quality score of both eyes	0.75 (0.48–1.18)	0.212	0.97 (0.57–1.65)	0.924

* OR standardized for age or age and gender. Abbreviations: PBBT: post-blink blur time; CCRLU: Cornea and Contact Lens Research Unit; LIPCOF: lid-parallel conjunctival folds; TBUT: tear break-up time.

## Data Availability

The authors confirm that the data supporting the findings of this study are available within the article and from the corresponding author upon request.
